# Membrane Profiling by Free Flow Electrophoresis and SWATH-MS to Characterize Subcellular Compartment Proteomes in *Mesembryanthemum crystallinum*

**DOI:** 10.3390/ijms22095020

**Published:** 2021-05-09

**Authors:** Qi Guo, Lei Liu, Won C. Yim, John C. Cushman, Bronwyn J. Barkla

**Affiliations:** 1 Southern Cross Plant Science, Faculty of Science and Engineering, Southern Cross University, Lismore, NSW 2480, Australia; q.guo.12@student.scu.edu.au (Q.G.); ben.liu@scu.edu.au (L.L.); 2 Department of Biochemistry and Molecular Biology, University of Nevada, Reno, NV 89557, USA; wyim@unr.edu (W.C.Y.); jcushman@unr.edu (J.C.C.)

**Keywords:** subcellular proteomics, membrane fractionation, membrane proteome, marker proteins, subcellular localization, lipid metabolism, lipid biosynthesis, ATPase, mass spectrometry, peptide library

## Abstract

The study of subcellular membrane structure and function facilitates investigations into how biological processes are divided within the cell. However, work in this area has been hampered by the limited techniques available to fractionate the different membranes. Free Flow Electrophoresis (FFE) allows for the fractionation of membranes based on their different surface charges, a property made up primarily of their varied lipid and protein compositions. In this study, high-resolution plant membrane fractionation by FFE, combined with mass spectrometry-based proteomics, allowed the simultaneous profiling of multiple cellular membranes from the leaf tissue of the plant *Mesembryanthemum crystallinum*. Comparisons of the fractionated membranes’ protein profile to that of known markers for specific cellular compartments sheds light on the functions of proteins, as well as provides new evidence for multiple subcellular localization of several proteins, including those involved in lipid metabolism.

## 1. Introduction

The plant membrane bilayer provides a barrier between cells and organelles and their surroundings, providing protection from a constantly challenging environment. Cellular membranes also allow compartmentalization of biochemical reactions and pathways into defined units, conferring specific organelles with distinct functions within the cell [1,2]. Despite their importance for this, the contribution and complexity of the broad range of lipids and proteins that make up the different membranes are far from fully understood, and especially, the underlying mechanisms that lead to the diversity of lipid composition remain to be explored [3,4]. A comprehensive analysis of the molecular composition of subcellular membranes would help to provide a more detailed understanding of their organization and function. While mass spectrometry-based proteomics has transformed the ability to perform large-scale protein identification, the ability to profile cellular organelles and endomembranes is still hampered by the limited techniques available to fractionate the membranes. Moreover, when doing this, it is important to be able to associate protein composition with lipid profiles when trying to explain the complexity in membrane processes.

In the past, plant researchers have tried to link the biological activity of membrane fractions with their lipid composition using fractionated subcellular membranes. However, data are generally restricted to studying a single organelle rather than providing a global cellular overview. Approaches such as Localization of Organelle Proteins by Isotope Tagging (LOPIT) have shown promise to decipher protein localizations based on subcellular fractionation by centrifugation and isotope tagging [5,6]. However, this method is unable to associate protein features with lipid profiles of subcellular membranes. Therefore, alternative approaches, which rely on distinct physicochemical properties of membranes while maintaining the structural integrity of lipids and proteins, are required.

**Figure 1:**
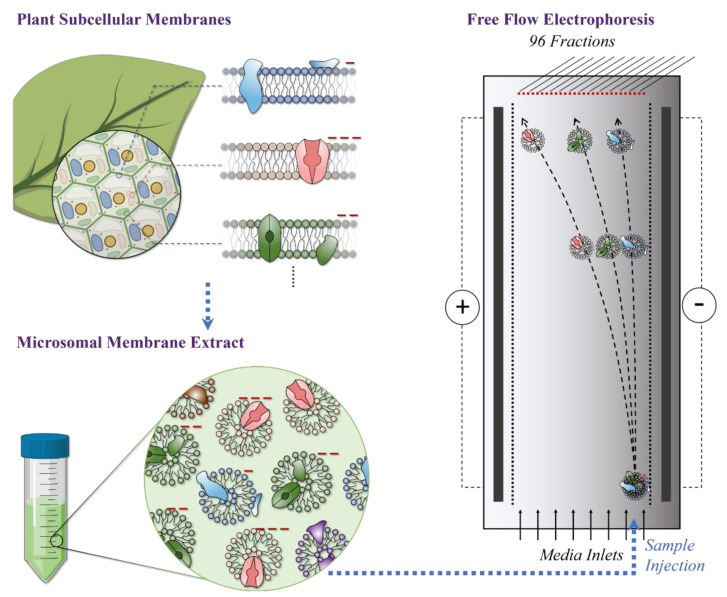
Schematic diagram of the working principle of Free Flow Electrophoresis (FFE) for plant subcellular membrane fractionation. Subcellular membranes with different origins (organelle/subcellular compartment) possess different overall surface charges mainly due to the chemical and compositional diversity of membrane lipids and proteins. In general, membranes exhibit negative surface charges around a neutral pH. Subcellular membranes form vesicles during the microsomal extraction procedure as described in “Materials and Methods”. Microsomal membranes are injected into the chamber and subjected to a high-voltage electric field. Membranes separate according to their distinct surface charge.

Free Flow Electrophoresis (FFE) allows for the continuous electrophoretic separation and fractionation of cells, organelles, membranes, and proteins from a wide variety of organisms and cell types in a thin, non-denaturing liquid film based on net surface charge [7]. The surface charge of subcellular membranes is the sum of mainly the protein and lipids’ combined charges [8] (Figure 1). High resolution and extreme reproducibility are the highlights of this technique [9,10,11,12,13,14,15,16,17,18,19,20,21]. FFE technique involves injecting a complex mixture of membranes that have been initially collected by ultra-centrifugation of cell lysates, into a chamber with a laminar flow buffer stream (Figure 1). These membranes are then deflected by a perpendicular electric field, with the degree of deflection directly related to the charge of the membrane. Fractions (up to 96) are then collected at the end of the separation chamber (Figure 1).

In plants, FFE has been used to increase the purity of specific organelle and endomembrane fractions by first isolating/purifying the organelle via conventional methods and then sub-fractionating those samples. In this way, FFE fractionation has been successfully achieved for thylakoid membranes, ER/Golgi membranes, peroxisomes, mitochondria, vacuole, and plasma membranes [22,23,24,25,26,27], but until now, this method has not been used as a method for global profiling across all cellular membranes in plants.

Combining the high-throughput, high-resolution FFE technology for fractionation of cellular membranes with LC-MS/MS proteomic approaches allows for the comparison of the abundance profiles of proteins throughout all separated fractions. These can then be compared to the profile of known marker proteins of specific compartments to yield classes of co-fractionated proteins from the same organelles, and can allow for the identification and characterization of subcellular compartments. Further, fractionated samples with known subcellular localization can be used for lipidomic studies. Additionally, with the knowledge of the locations of lipid metabolism-related proteins, subcellular metabolic maps of plant lipids may be achieved.

As a model plant for the study of salinity in plants, *Mesembryanthemum crystallinum* employs both structural and metabolic adaptations including the presence of epidermal bladder cells for salt accumulation and shifting from C3 photosynthesis to CAM to increase water use efficiency [28]. Additionally, at the molecular level, salinity has been show to induce changes to membrane lipids and proteins involved in transport, signaling and compatible solute synthesis [29,30,31]. In this study the ability to fractionate subcellular membranes of *M. crystallinum* and identify proteins and lipids will facilitate our understanding of the importance of membrane regulation and remodeling in salinity tolerance.

## 2. Results

### 2.1. M. crystallinum Membrane Proteome

**Figure 2:**
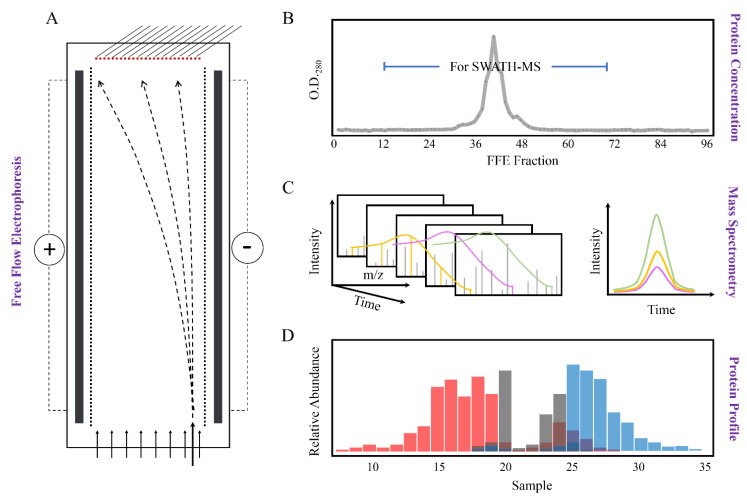
Schematic overview of sample fractionation by Free Flow Electrophoresis (FFE) and downstream analysis by mass spectrometry. (**A**) Microsomal membranes from *M. crystallinum* leaf tissue were fractionated by FFE into 96 fractions based on their different net surface charge; (**B**) The absorbance of FFE fractions was determined at 280 nm to identify the range of fractions with positive protein values. Fractions 15 to 70 with positive protein values at O.D._280_ were used for subsequent protein identification; (**C**) Proteins were analyzed by SCIEX TripleTOF 6600 in IDA and SWATH-MS modes for reference library generation, protein identification, and quantification; (**D**) The average protein abundance of three biological replicates of each sample was used for the “digital western” FFE profile generation. Here we use the term digital western to refer to the detection of proteins by MS/MS in specific FFE fractions, similar to a traditional Western where proteins in the wells of a gel are detected by antibodies. Different colored columns indicate different subcellular membrane origins based on marker protein profiles.

Free Flow Electrophoresis of microsomal membranes from leaf tissue of *M. crystallinum* plants resulted in the high-resolution fractionation of membranes into 96 samples. Every second fraction was then combined to give a total of 48 protein samples (i.e., sample 1 = FFE fractions 1 and 2, sample 2 = FFE fraction 3 and 4, etc.). Fractions 15 to 70 (combined samples 8 to 35) with positive protein values at O.D._280_ (Figure 2B) were subsequently analyzed by IDA for ion library generation and SWATH-MS for protein quantification. A schematic representation of the approach is illustrated in Figure 2.

Spectra acquired by information-dependent acquisition (IDA) from all samples (28 samples × 3 biological replicates) were submitted to ProteinPilot 5.0.2 to generate the reference spectral library, which included the fragmentation patterns and retention time of each peptide that was required for targeted identification in SWATH-MS [32]. Peptides with confidence scores higher than 95% were selected and summarized. The final ion library contained 1917 distinct proteins at a critical false discovery rate of 1%. The identification and quantification of proteins were performed using a SWATH-MS approach, which resulted in the quantitative export of 1462 unique proteins across all the FFE samples.

**Figure 3:**
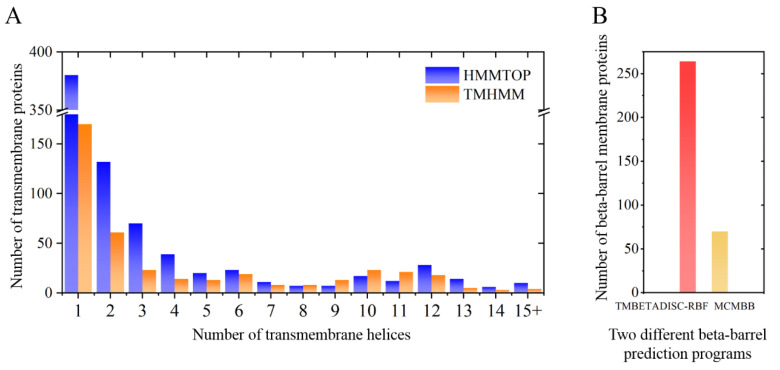
Number of transmembrane domains predicted in the *M. crystallinum* membrane proteome. (**A**) Transmembrane helix prediction by HMMTOP and TMHMM, and (**B**) beta-barrel predictions by TMBETADISC-RBF and MSMBB transmembrane prediction data are summarized in Table S1.

In order to describe the composition of the *M. crystallinum* membrane proteome, transmembrane domains were predicted using the transmembrane topology (HMMTOP v2.0 and TMHMM v2.0) and beta-barrel membrane (MCMBB and TMBETADISC-RBF) prediction programs [33,34,35,36]. The results are shown in Table S1. Proteins predicted to possess a transmembrane domain by any of the four prediction programs (Figure 3) were considered as membrane proteins. Over 62% (908 out of 1462) of the *M. crystallinum* membrane proteome could be assigned to integral membrane proteins with at least one transmembrane domain, while the others were considered as peripheral or membrane-associated proteins.

### 2.2. Functional Annotation and Subcellular Localization

**Figure 4:**
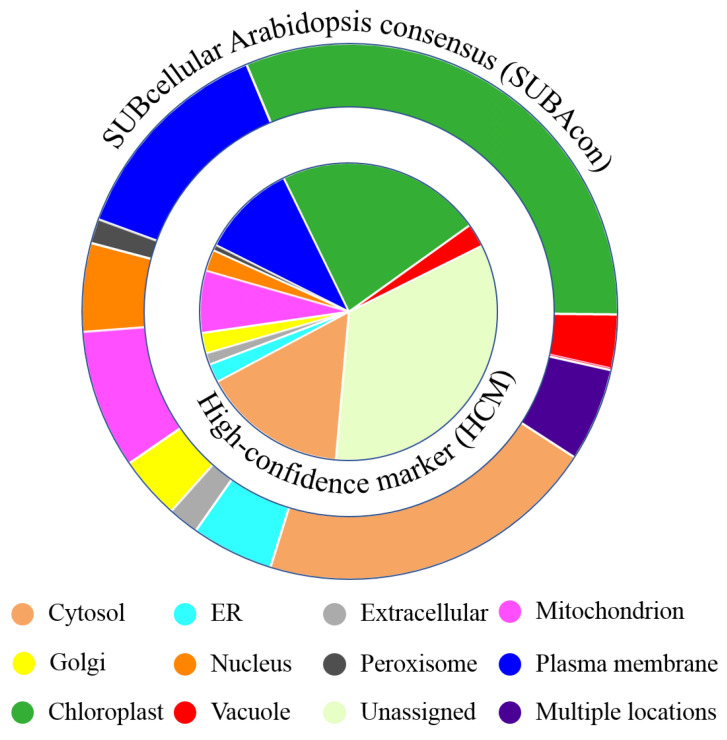
Percentage of the number of distinct proteins for each compartment by (inner pie chart) high-confidence markers (HCM) assignment and (outer pie chart) SUBcellular *Arabidopsis* consensus (SUBAcon) predictions.

Functional annotation of the identified proteins was based on the best BLASTP hits found in the National Center for Biotechnology Information (NCBI), UniprotKB/SwissProt *A. thaliana* database (May 2020), with 10^−5^ as the e-value cut-off. The majority (1290, ~88%) of the proteins were matched to homologs from *A. thaliana* (Table S2). The matched *A.* *thaliana* genome accessions (AGIs) were then submitted to the Subcellular Localisation Database For *Arabidopsis* Proteins 4 (SUBA4) [37] for subcellular localization information. According to SUBA4, approximately 66% of the 1290 proteins were assigned to a high-confidence marker (HCM) for subcellular compartments (Table S2), and the percentage of proteins assigned to each compartment is displayed in the pie graph in Figure 4. Disregarding the unassigned category, proteins classified to a chloroplast HCM comprised the largest proportion of the *M. crystallinum* membrane proteome (22.3%), followed by cytosol (15.8%), plasma membrane (10.4%), and mitochondria (6.7%) (Table S2). The secretory pathway (from the ER to the TGN) together accounted for 4.3%, while the remaining categories were less than 3%.

Although abundant experimental data from fluorescent protein tagging or mass spectrometry are available for the *A. thaliana* proteome, only approximately 30% of the proteome is covered by high-confidence location data, whereas the remaining 70% has only been computationally predicted [38]. Using a naive Bayes classifier, the SUBcellular *Arabidopsis* consensus (SUBAcon) algorithm integrates 22 computational prediction algorithms, for experimental GFP and MS localization, protein–protein interactions, and co-expression data to derive a consensus location of proteins [38]. The SUBAcon predictions of the submitted proteome are listed in Table S2. Based on these predictions, 31.5% of the *M. crystallinum* leaf proteome were categorized as chloroplast proteins, while 20.7 and 13.1% were from cytosol and plasma membrane, respectively (Figure 4). Proteins assigned to other locations accounted for less than 10%, with 8.7, 8.4, and 5.3% classified to the secretory pathway, mitochondria, and nuclear proteins, respectively. The remaining categories comprised less than 5%, with vacuolar and peroxisome proteins representing 3.3 and 1.5%, respectively. Notably, 5.7% of the identified proteome was predicted to be present in multiple subcellular locations (Figure 4, Table S2).

### 2.3. FFE Profile of Representative Subcellular Membrane Markers

The averaged abundance of proteins from three biological replicates across all analyzed FFE samples was then carried out to obtain “digital westerns” (Figure 2D), whereby peak area for representative well-characterized marker proteins present in the samples was aligned and visualized in Table 1, demonstrating the positioning of specific subcellular membranes. These markers have been previously verified experimentally with high confidence using cell biology approaches and are accepted to be representative of their particular compartment [37].

Vacuole membrane (tonoplast)-associated proteins appeared in two distinct populations (Table 1 and Table 2). The first population eluting at the farthest left of the FFE chamber showed the highest mobility moving towards the anode electrode from samples 13 to 19; the second population, which showed reduced mobility, was observed as a smaller population with a peak at sample 24. The FFE profiles of the tonoplast H^+^-ATPase subunits exhibited isoform- and subunit-specific patterns. For example, VHA-B2, VHA-E3, and VHA-F were present evenly in both populations, while the abundance of VHA-a3, VHA-c2, and VHA-d2 were much higher in the anodic population (Table 2). Surprisingly, VHA-A and VHA-D were mainly identified in the second population with a peak at sample 24 and were barely detected in samples 13 to 19. Similar to VHA-a3, VHA-c2, and VHA-d2, tonoplast aquaporins and ion channels, including delta-TIP (TIP2-1) and gamma-TIP (TIP1-1), two-pore calcium channel protein 1 (TPC1), chloride channel protein B (CLC-B), and CLC-C were also present in both populations (Table 2), despite the peak of the second population being much smaller compared to their anodic populations (Table 2).

ER membrane markers, represented by cytochrome b5 isoform B (CYTB5-B) and very-long-chain 3-oxoacyl-CoA reductase 1 (KCR1) (Table 1), were present in two populations from samples 18 to 20, and 23 to 25 (Table 3). Markers of the Golgi apparatus overlapped with ER markers, although the protein abundance was relatively greater in the second population representing samples 23 and 24, as shown by the profiles for polygalacturonate 4-alpha-galacturonosyltransferase (GAUT1) and probable methyltransferase PMT8 (At1g04430) in Table 3. Notably, proteins that are known to frequently exchange between the ER and Golgi were in very low abundance in the anodic population. As an example, Coat Protein Complex II (COPII)-associated Sec23/Sec24, and COPI-associated coatomer subunits, were found almost exclusively in samples 23 and 24 (Table 3). In addition, post-Golgi trafficking markers, shown as AP-1 complex subunit gamma-2 and clathrin heavy chain 1, were mostly concentrated in a single FFE sample, 24, suggesting a slightly different profile of these proteins from those exchanged between ER and Golgi.

Proteins localized to chloroplast envelope membranes, such as inner/outer envelope membrane protein translocon at the inner envelope membrane of chloroplasts 110 (TIC110) and translocon at the outer envelope membrane of chloroplasts 34 (TOC34), were present in a single population fractionating to the middle of the FFE chamber from samples 23 to 25 (Table 1). Nevertheless, proteins localized to thylakoid additionally presented in high abundance in sample 20, as was observed for ATP synthase gamma chain 1 (ATPC1), ATP synthase subunit delta (ATPD), and light reaction-related proteins, with only two exceptions found for photosystem I (PSI) reaction center subunit II-2 (PSAD2) and subunit psaK (PSAK) which presented almost exclusively in FFE samples 21 and 22 (Table 4).

Proteins with known localization to mitochondria were also found in sample 20 (Table 5), although their relative abundance in sample 20 was dramatically lower than that of thylakoid markers (Table 4), as is shown for the mitochondrial outer membrane protein porin 1 (VDAC1) and ATP synthase subunit beta-3 (mitochondrial inner membrane localized) (Table 1 and Table 5). It is also interesting to find that alternative NAD(P)H-ubiquinone oxidoreductase C1 (NDC1), which was previously shown to have dual subcellular locations in both chloroplast and mitochondria [39], has a slightly different profile than either chloroplast or mitochondrial markers in the FFE fractions, with a higher abundance than mitochondrial markers in a population with a peak at sample 20, and also a wider distribution (samples 23–26) than that of the cathodic population of the chloroplast markers which were present mostly in samples 23 and 24 (Table 5).

The PM was the least negatively charged subcellular compartment, fractionating closest to the cathode during FFE. The FFE profile of the PM was determined by the presence of plasma membrane marker proteins in FFE samples 24 to 30 with a peak of abundance at sample 25, for instance, P-type H^+^-ATPase including AHA2, AHA4, and AHA5, as well as specific PM water channel proteins such as MIP-A, MIP-D, MIP-H, and PIP2;8, with only the profile of PIP2;5 slightly moved towards the anode electrode with a peak of abundance at sample 24 (Table 1 and Table 6). PM-localized transporters, such as sucrose transport protein SUC2 and polyol transporter 5 (PLT5), also showed a typical protein distribution in line with other PM markers. Worth mentioning, as previously reported, is PM nanodomain-localized proteins, fasciclin-like arabinogalactan proteins 2 and 6 (FLA2 and FLA6), and early nodulin-like protein 2 [40], distributed within a relatively narrower population, were only identified in samples 24 to 26 (Table 6).

Table 7 summarizes the distribution and corresponding abundance of several proteins which show unexpected location profiles in the samples, which do not match the published reports. These include sodium/hydrogen exchanger 7 (NHX7/SOS1) [41], sodium/calcium exchanger (NCL) [42], ammonium transporter 1 member 1 (AMT1-1) [43], ABC transporter G family member 40 (ABCG40) [44], ABC transporter C family member 4 (ABCC4) [45], and equilibrative nucleotide transporter 1 (ENT1) [46]. According to previous experimental evidence and SUBAcon, these proteins are suggested to be PM localized. However, in this study, they all fractionated into anodic FFE samples 13-19, showing the greatest overlap with tonoplast markers. Indeed, there are previous reports that have experimentally provided evidence for the dual location of two of these, ABCC4 and ENT1, which were both shown to localize to TP and PM [45,46,47,48]. 

### 2.4. FFE Profiles of Lipid Metabolism-Related Proteins

With the help of “digital westerns” constructed using well-characterized membrane marker proteins that revealed the separation and distribution of subcellular compartments across the FFE fractions, a map of the distribution of lipid metabolism-related proteins can be generated. Protein profiles of these proteins are shown in Table 8. The predicted localization of the listed proteins indicates, as expected, that lipid metabolism occurs mainly in Golgi, ER, PM, and chloroplast (Table 8), and these proteins can be assigned to pathways including fatty acid synthesis and elongation, galactolipid and sulfolipid synthesis, phospholipid synthesis and signaling, triacylglycerol synthesis, sterol synthesis, and suberin synthesis.

A good overlap of the protein profiles of lipid metabolism-related proteins with marker proteins from the expected subcellular compartments was observed from the results. For example, lipid-related proteins suggested as chloroplast membrane localized, such as esterase lipase thioesterase family protein, trigalactosyldiacyl-glycerol 2 and 4 (TGD2 and TGD4), fatty acid desaturase 6 and 8 (FAD6 and FAD8), alpha beta-hydrolases superfamily protein (DALL3), single hybrid motif superfamily protein (BCCP2), and long-chain acyl-CoA synthetase 9 (LACS9), were present mainly in the single fraction sample 20 and in the population fractionating to the samples 23 and 24, which match the protein profiles of chloroplast markers present in Table 4. However, the protein abundance of these proteins in the single chloroplast fraction at sample 20 was much lower than was observed for fractions 23 and 24. Moreover, phospholipid glycerol acyltransferase family protein (ATS1/GPAT), AMP-dependent synthetase, and ligase family protein (AAE16) were completely absent from that fraction. The protein distribution for acetyl CoA carboxylase carboxyltransferase alpha subunit (CAC3), however, was different from the other chloroplast-localized proteins, as it was also identified in FFE fraction samples 15 to 19.

Proteins identified as ER proteins, such as beta-ketoacyl reductase 1 (KCR1), cinnamate-4-hydroxylase (CYP73A5), cytochrome b5 (CYTB5-B), phospholipid: diacylglycerol acyltransferase 1 (PDAT1), membrane-associated progesterone-binding protein 3 (MSBP2), 7-dehydrocholesterol reductase (DWF5), and very-long-chain enoyl-CoA reductase (ECR) all presented primarily in two distinct populations (from samples 18 to 20, and 23 to 25), matching what was observed for ER marker proteins (Table 8). Nevertheless, an exception was observed for synaptotagmin-1 (SYT1), which was previously classified as both an ER- and PM-localized protein [49,50]. In this study, the profile of SYT1 matched that of PM markers, being identified in samples 24 to 29 (Table 1 and Table 6), similar to other PM-localized lipid metabolism-related proteins (Table 8), including non-specific phospholipase C4 (NPC4), PLC-like phosphodiesterases superfamily protein, phospholipase D delta (PLDDELTA), non-specific lipid transfer protein GPI-anchored 2 (LTPG2), and phosphatidylinositol-specific phospholipase C4 (PLC4). Additionally, phosphatidylinositol 3- and 4-kinase family protein (PI4KA1), AMP-dependent synthetase, ligase family protein (LACS4), 3-ketoacyl-CoA synthase 6 (CUT1), ABC-2 type transporter ABCG15, and ABC-2 type transporter ABCG22 were also suggested as PM-localized lipid-related proteins by SUBAcon. However, these latter proteins were also identified in samples 13 to 19 overlapping with the tonoplast and the ER profiles, suggesting there may be uncharacterized subcellular locations and biological functions of these proteins.

## 3. Discussion

The ability to fractionate subcellular membranes with high resolution can provide important information for understanding the subcellular location and biological function of a protein [52]. Combining high-resolution membrane fractionation by Free Flow Electrophoresis (FFE) with the unbiased quantification method of SWATH-MS in this study allowed the characterization of protein profiles for multiple membrane compartments. Subcellular localization of numerous membrane proteins from *M. crystallinum* leaf tissue was confirmed by their FFE profiles and shown to overlap with known marker proteins. These results also enabled the identification of novel localizations for proteins outside their characterized resident membrane, providing insight into poorly characterized compartments.

### 3.1. V-ATPase VHA Subunit Localization

The vacuolar proton-pumping V-ATPase consists of 14 subunits divided between two distinct domains; the membrane integral V0 domain and the membrane peripheral V1 domain. In this study, we identified 11 subunits (VHA-A, VHA-B, VHA-C, VHA-D, VHA-E3, VHA-F, VHA-G1, VHA-H, VHA-a3, VHA-c2, and VHA-d2-). The majority of these had similar FFE profiles, showing two distinct populations (Table 2). The more negative anodic population showed close overlap with other tonoplast proteins, including pyrophosphate-energized vacuolar membrane proton pump 1 (AVP1), two tonoplast intrinsic proteins, aquaporins TIP1-1, TIP2-1, and the TPC1 calcium channel. However, there were several VHA subunits that only presented in one of these two populations.

Evidence has shown that V-ATPase subunits are localized not only to the tonoplast but also to the TGN in plants [53]. Previous proteomics studies have identified VHA-A subunit isoforms on Golgi in *A. thaliana* cell culture proteomic studies [54]. Moreover, pollen grains from an *A. thaliana vha-A* mutant exhibited severe alterations in the morphology of Golgi stacks and Golgi-derived vesicles, while vacuole morphology remained unaffected [55]. In a vha-a2/vha-a3 double mutant, which showed increased sodium sensitivity, reduction in V-ATPase activity in the trans-Golgi network/early endosome (TGN/EE) was observed, suggesting a role for the V-ATPase in salt tolerance in these membranes [53]. In this study, VHA-A and VHA-D appeared to be absent from the anodic population (samples 13–19), presenting almost exclusively in sample 24 (Table 2). The single population of these two V-ATPase subunit isoforms had almost identical FFE profiles to that of Golgi-specific markers, such as GAUT1, GAUT9, and PMT8 (Table 1, Table 2 and Table 3), supporting their Golgi localization and suggesting that these subunits may have a specific function in this membrane.

In contrast, the VHA-a3 subunit presented mostly in the anodic population from samples 13 to 19, suggesting it was solely tonoplast localized (Table 2), and indicated a different localization to that observed for the isoform in *A. thaliana* [56]. Additionally, the relatively higher protein abundance (by peak area) of the cathodic population of subunits VHA-B2, VHA-E3, VHA-F, and VHA-H suggested these may also play a role in the Golgi/TGN V-ATPase holoenzyme organization in *M. crystallinum* [57]. Previous MS/MS-based experiments have also shown their alternative subcellular locations on Golgi-related compartments [54,58].

### 3.2. Proteins with Unexpected FFE Profiles

The FFE profiles of several PM-classified transporter proteins were unexpected, with proteins exhibiting a fractionation profile overlapping with vacuolar markers, including sodium/hydrogen exchanger 7 (SOS1/NHX7), sodium/calcium exchanger (NCL), ammonium transporter 1-1 (AMT1-1), sulfite exporter TauE/SafE family protein 4 (TauE), ABC transporters (ABCG40 and ABCC4), and equilibrative nucleotide transporter 1 (ENT1) (Table 7). Among these proteins, SOS1, a well-studied member of the Na^+^/H^+^ exchanger (NHX7) family, shown to have an important role in plant salt tolerance, showed a profile similar to vesicle trafficking-related proteins, such as PRA1F2, RABB1C, RABG3A, and RABG3F (Table S3). While this protein has been characterized as functioning at the PM in plants [41,59], a study characterizing a *sos1* mutant in *A. thaliana* demonstrated multiple consequences in the cell due to the lack of this transporter, distinct from a role solely in plasma membrane sodium transport; these included the inhibition of vesicle trafficking and endocytosis in the mutant as well as a disintegrated tonoplast and a decrease in vacuolar pH [60]. Further evidence for a non-PM role was shown in another study which demonstrated that the function of the vacuolar-type H^+^-PPase in *A. thaliana* under salt stress required the regulation of SOS1, as the pyrophosphate-dependent proton pump activity of the vacuolar-type H^+^-PPase was significantly lower in the AVP1/sos1 line compared with AVP1 overexpression line, suggesting SOS1 is epistatic to AVP1 [61]. However, evidence for direct interaction between these two proteins was not presented.

In addition, while NCL and AMT1-1 are accepted as PM-localized proteins involved in the transport of sodium/calcium and ammonium, respectively [42,43,62], evidence for non-PM localization has been presented, including vacuolar localization of these proteins as shown by fluorescent-tagging and MS/MS-based approaches [48,63,64], although the biological function for their role on the tonoplast has not been determined. Similar to what was shown for SOS1 and mentioned above, FFE fractionation may have also captured these proteins in an endomembrane trafficking compartment as they share a similar profile to a number of small GTPases (Table S3).

Our study also captured a number of mitochondrial proteins that appeared to be co-localized with plasma membrane fractions, such as cytochrome b-c1 complex subunit Rieske-1 (UCR1-1), mitochondrial-processing peptidase subunit beta (MPPbeta), and mitochondrial carnitine/acylcarnitine carrier-like protein (BOU) (Table 5 and Table 6). Mitochondrial membranes have been shown to be tethered to the PM in yeast [65] and have been identified in plasma membrane proteomics studies in plants, including a recent study using highly purified plasma membrane fractionated in tandem by two-phase partitioning, followed by FFE [26]. This suggests that rather than contaminants, they may represent mitochondrial proteins associated with the plasma membrane through specific contact points. As FFE is a non-denaturing technique, it is highly likely that membrane interactions remain intact during the fractionation.

### 3.3. A Snapshot of the Components of Electrochemical Reactions

The FFE fractionation of photosynthetic membranes was also observed in this study. Photosynthesis occurs on the thylakoid membrane inside the lumen of the chloroplast, enclosed by a double-membrane envelope composed of the outer envelope membrane (OEM) and the inner envelope membrane (IEM) [66]. The separation of OEM and IEM was not observed in this study, as the OEM marker TOC34 and IEM markers, TIC55 and TIC110, were all mostly present in samples 23 and 24.

However, while the chloroplast inner and outer membranes fractionated together in samples 23 and 24, a particularly large number of thylakoid membrane proteins were identified in sample 20 (Table 4). The thylakoid membrane is a lipoprotein system that has an inner and outer surface with the inner face being more negatively charged [67], and might explain the two distinct populations of thylakoid membrane markers in this study (Table 4). Barber [67] has suggested that the thylakoid membrane is not homogeneous and is likely to be derived mainly from protein rather than lipid components. Interestingly, the protein profile of the primary electron acceptor of photosystem I, ferredoxin-1 [68] was observed to be highly negatively charged in this study (Table 9).

Ferredoxin-1, which based on sequence analysis does not possess any transmembrane domains (tr_41050, Table S1), is likely associated with the membrane via protein/protein interactions with the PS1 trimer. In support of this, a recent study using affinity chromatography and nuclear magnetic resonance demonstrated that the trimeric PSI complex binds three ferredoxins, but in a non-equivalent manner, with one ferredoxin showing stronger binding to PSI than the other two [69]. This might explain our results with the negatively charged population of ferredoxins in the chamber (samples 8 to 14) representing unbound protein that has been released from its protein interaction with PS1 due to the FFE conditions and is more strongly attracted to the anodic electrode during FFE, while the small population found in samples 23 and 24 represents protein still firmly bound to the PSI on the thylakoid membrane, as shown by the small population of this protein which overlapped with thylakoid profiles (Table 9). Interestingly, this is the only protein that shows this profile, and no other proteins were detected in samples 8 to 14 in this study.

### 3.4. Subcellular Mapping of Lipid Metabolism

Characterization of the membrane distribution of lipid metabolism-related proteins may give a better understanding of lipid signaling, as well as the compositional variance in lipids within subcellular membranes, and how these change under certain developmental and environmental conditions. While previous efforts have demonstrated lipid metabolism pathways based on the types of lipids produced and their subcellular locations [51], the exact localization of some of the enzymes in the biosynthetic pathways remained under question, or in some cases were identified in different subcellular compartments by different studies [70]. The comparative analysis of the protein profiles of lipid metabolism-related proteins with well-known markers in this study would help provide a means for more precise localization of these proteins.

Lipid biosynthesis enzymes are often promiscuous, utilizing a broad range of more or less similar substrates, and the production of these substrates is usually strictly spatially compartmentalized within cells [3], making it easier for us to observe the biological importance of each pathway. To give an example, the plastid is the main site for fatty acid synthesis and glycerolipid production in plants and algae [70,71,72]. Our results agree with this, as lipid biosynthesis proteins suggested as plastid localized by SUBAcon, present mainly in sample 20 and in the population fractionating in samples 23 to 25 (Table 8), matching the profiles of chloroplast markers shown in Table 1 and Table 4. However, unlike what was observed for thylakoid lumenal protein, chlorophyll a-b binding proteins, and photosystem I/II reaction center subunits (Table 4), lipid metabolism-related proteins (chloroplast localized) had much lower abundance in sample 20 (Table 8). This is likely due to the fact that biosynthesis of plastid glycerolipids takes place in the envelope membranes, which is the site of fatty acid assembly [71,73,74], whereas the build-up of thylakoid membranes requires the transport of lipids from envelope membranes during plastid biogenesis [75,76]. Notably, different from the other chloroplast-localized proteins, acetyl CoA carboxylase carboxyltransferase alpha subunit was localized to samples 15-19 (Table 8). Although this protein is a high-confidence marker for chloroplasts, it was previously observed in Golgi [58], vacuole [77], and plasma membrane [78] by MS/MS approaches, indicating this protein may have uncharacterized subcellular locations and biological functions.

In addition to the plastid (prokaryotic pathway), the ER is the other main site for fatty acid elongation, acyl editing, and lipid assembly through the eukaryotic pathway. It was suggested that in *A. thaliana*, around 2/3 of the fatty acids are thought to be exported to the eukaryotic pathway, although half of which will be returned for plastid lipid assembly [79]. Our data show that proteins suggested as being involved in the fatty acid synthesis and elongation distributed to the same FFE fractions as the ER markers (Table 3 and Table 8), fractionating in samples 18 to 20, and from 23 to 25.

The ER-type fractionation profiles were also seen for proteins that participate in other ER-localized lipid metabolism pathways, such as triacylglycerol biosynthesis (phospholipid: diacylglycerol acyltransferase) and steroid metabolism (membrane-associated progesterone-binding protein 3 and 7-dehydrocholesterol reductase). Notably, the protein abundance in samples 25 to 28 for phospholipid: diacylglycerol acyltransferase, membrane-associated progesterone-binding protein 3,7-dehydrocholesterol reductase, and very-long-chain enoyl-CoA reductase family protein was relatively high compared to the other ER-localized proteins. Interestingly, these proteins were also identified as abundant plasma membrane-localized proteins in previous studies using MS/MS proteomic approaches [26,78,80,81], indicating their subcellular location may need to be redefined and re-validated. By contrast, concrete evidence by fluorescent protein methods has shown the dual localization at both ER and PM of the lipid-binding protein synaptotagmin-1 [49,50], whereas its protein profile is more like that of PM markers in this study (Table 6 and Table 8).

Lipid metabolism-related proteins, including non-specific phospholipase C4, PLC-like phosphodiesterases superfamily protein, phospholipase D delta, non-specific lipid transfer protein GPI-anchored 2, and phosphatidylinositol-specific phospholipase C4, were suggested as PM-localized proteins by SUBAcon and in this study presented in samples 24 to 29 with a peak at 25, which matched the protein profiles of PM markers (Table 6 and Table 8). Nevertheless, several proteins, such as phosphatidylinositol 3- and 4-kinase family protein, AMP-dependent synthetase and ligase family protein, lipid phosphate phosphatase 2, 3-ketoacyl-CoA synthase 6, and ABC-2 type transporter family proteins ABCG15 and ABCG22 were present in samples 13 to 19. Notably, even though 3-ketoacyl-CoA synthase 6 was suggested as PM localized by SUBAcon, it has been hypothesized that this protein is likely involved in the transport of lipids (wax) from the ER to the PM [82], while further verification of its ER localisation has been provided by fluorescent microscopy [83]. In agreement with this, our data also showed the ER marker-like profile of this protein, demonstrating the sensitivity of the method used in this study to characterize protein subcellular location. Furthermore, evidence of non-PM locations was also available for other proteins (suggested as PM localized by SUBAcon). For example, phosphatidylinositol 4-kinase was observed in the cytosol by fluorescent protein assay [84] and AMP-dependent synthetase and ligase family protein was detected in vacuole and trans-Golgi network by MS/MS methods [58], suggesting this approach can also provide valuable information on proteins located in multiple cellular compartments.

## 4. Materials and Methods

### 4.1. M. crystallinum Plant Material

*Mesembryanthemum crystallinum* L. seeds were sown on commercial soil mix supplemented with dolomite, slow-release fertilizer (Scotts Osmocote^®^, Bella Vista, NSW, Australia), and micronutrients (Micromax^®^, Unanderra, NSW, Australia) in a seedling propagation tray. Three-week-old seedlings of uniform size were transplanted to pots containing the same soil mixture, with two plants per 15 cm diameter pot. Plants were watered daily and supplemented with one-half strength Hoagland’s solution weekly. Once the plants had reached eight weeks old, the second pair of mature leaves were collected for downstream experimental analysis. Plants were grown in a glasshouse with evaporative cooling to maintain the temperature between 15 and 32 °C and under natural irradiation and photoperiod (photon flux density maximum of 1300 mmol m^−2^ s^−1^).

### 4.2. Microsomal Membrane Extraction

Leaf material (60 g) from *M. crystallinum* was harvested, cut into small pieces, and immediately placed into 300 mL of ice-cold homogenization medium consisting of 400 mM mannitol, 10% (*w*/*v*) glycerol, 5% (*w*/*v*) polyvinylpyrrolidine-10, 0.5% (*w*/*v*) bovine serum albumin, 1 mM phenylmethylsulphonyl fluoride, 2 mM dithiothreitol, 30 mM trisaminomethane, 5 mM MgSO_4_, 5 mM EGTA, 0.5 mM butylated hydroxytoluene, 0.25 mM dibucaine, 1 mM benzamidine, and 26 mM K^+^-metabisulfite, adjusted to pH 8.0 with H_2_SO_4_. Once homogenized in a commercial blender, leaf tissue was filtered through cheesecloth and concentrated by centrifugation in a Beckman L8-M ultracentrifuge at 100,000× *g* for 50 min at 4 °C. The supernatant was removed, and microsomal pellets were resuspended using a Teflon pestle in suspension buffer containing 400 mM mannitol, 10% (*w*/*v*) glycerol, 6 mM Tris/Mes (pH 8.0), and 2 mM dithiothreitol, then immediately frozen in liquid nitrogen for storage at −80 °C.

### 4.3. Free Flow Electrophoresis (FFE)

Microsomal membranes (3 biological replicates) from leaf tissue of *M. crystallinum* were fractionated using a Free Flow Electrophoresis System (FFE Service GmbH). Samples were diluted 2:1 (*v*:*v*) in separation medium containing 250 mM sucrose, 10 mM acetic acid, 10 mM triethanolamine, and 2 mM KCl, and centrifuged at 14,000× *g* for 20 min at 4 °C prior to injection into the FFE chamber. Diluted samples (3 mg/mL protein) were injected continuously through a peristaltic pump at a rate of 1.2 mL/h via the anodic sample inlet while media inlet composition was as follows: inlets 1 and 9, stabilization medium comprising 180 mM sucrose, 40 mM acetic acid, 40 mM triethylamine, and 8 mM KCl; inlets 2 to 6, separation medium comprising 250 mM sucrose, 10 mM acetic acid, 10 mM triethylamine, and 2 mM KCl: cathodic and anodic circuit electrolyte solutions consisted of 100 mM triethylamine, 100 mM acetic acid, and 10 mM KCl adjusted to pH 7.4 with NaOH. In order to avoid the loss of chloride by anodic oxidation, formaldehyde (0.4%) was added to the anodic solution. The counter flow medium was the same as the separation medium for inlets C1, C2, and C3. FFE was conducted in a horizontal mode at a constant voltage of 750 V (~135 mA) with a media and counterflow rate of 250 mL/h. The temperature of the chamber was maintained at 5 °C throughout the whole FFE process by the continual flow of coolant from a circulating water bath. Each injected sample was fractionated into 96 fractions, which were then continually collected into 15 mL polypropylene tubes placed on ice. Fractionation by FFE was monitored by collecting microtiter plates (200 mL/well) at the beginning and end of each run and protein in each well measured by taking the O.D._280_ reading using a microplate scanning spectrophotometer (Victor X4 2030 Multilabel Reader, PerkinElmer, Turku, Finland). Every second fraction was then combined to give a total of 48 protein samples (i.e., fractions 1 and 2, 3 and 4, 5 and 6, etc.). Samples from numbers 8 to 35, which gave positive protein values at O.D._280_, were used in subsequent applications.

### 4.4. TCA Precipitation and Trypsin Digestion of Membrane Proteins

Two hundred microliters of 10X TE, 0.3% (*w*/*v*) sodium deoxycholate, and 72% (*w*/*v*) trichloroacetic acid were added to 1 mL of each sample in a centrifuge tube in sequence and vortexed between every step. Samples were incubated on ice for 1 h, and the supernatant was discarded by aspiration after centrifugation in a Sigma 4K15 laboratory centrifuge at 14,000× *g* for 20 min at 4 °C. Pellets were resuspended with 90% methanol then incubated overnight at −20 °C. Following the repeated steps of centrifugation and aspiration, protein pellets were dried in a fume cabinet on ice.

Samples were made up to 1 mg/mL concentrations using 2 M urea in 50 mM ammonium bicarbonate (pH 8.0). One hundred microliters of each sample were digested with 20 µg trypsin and incubated in an ultrasonic bath for 10 min. Tryptic digestion was carried out overnight at 37 °C followed by 4 min in a microwave on the lowest power setting. Samples were dried down using a Heto vacuum centrifuge at 45 °C for 2 h or until dry and dissolved in 100 µL 1% trifluoroacetic acid in milliQ water, then 50 µL samples were aliquoted into autosampler vials for injecting into the nanoHPLC/MS MS/MS system.

### 4.5. Information-Dependent Acquisition (IDA)

The tryptic peptide samples were analyzed using Ekspert nano LC400 uHPLC (SCIEX, Concord, ON, Canada) coupled with a TripleTOF^®^ 6600 quadrupole time-of-flight (QTOF) mass analyzer (SCIEX, Canada) equipped with a PicoView nanoflow (New Objective, Woburn, MA, USA) ion source. A trap column (5 mm × 300 µm, C18 3 µm, SGE, Ringwood, VIC, Australia) and an analytical column (75 µm × 150 mm ChromXP C18 CL 3 µm, SCIEX, Canada) were used. A linear gradient consisting of 0.1% formic acid in water (Solvent A) and 0.1% formic acid in acetonitrile (Solvent B) was employed with Solvent B from 2 to 40% over 60 min, 40 to 90% for 5 min, 90% for 5 min, and equilibration at 2% between samples. The mobile phase flow rate was 400 nL/min, and the column temperature was set at 45 °C. The peptide samples (5 µL each) were loaded onto the trap column (10 µL/min, 5 min), and eluted onto the analytical column at 400 nL/min. The ionization parameters were as follows: interface heater 150 °C, ionspray voltage 2600 V, declustering potential 80 V, and both nebulizer gas 1 (GS1) and curtain gas flow values set at 30. The protein identification for peptide library production was achieved using the information-dependent acquisition (IDA) mode of the QTOF. During the analysis, a full scan TOF (50 ms, 350–1800 m/z) was first acquired, and then the 30 most intense ions (with minimum 200 counts and a charge state of +2 to +5) of the TOF scan were selected to generate MS/MS spectra (100 ms each, with a rolling collision energy, product ion scan 100–1500 m/z) using the QTOF. Analyst TF 1.7 software (SCIEX, Canada) was used for data acquisition and analysis.

### 4.6. Sequential Window Acquisition of All Theoretical Mass Spectra (SWATH-MS)

Peptide and protein quantification data were acquired under the same HPLC and mass spectrometry experimental conditions as described for IDA, with the following exceptions. Data were acquired using a SWATH, product ion ms/ms all approach, which continuously acquires all fragment ion spectra (MS2) in an unbiased fashion for every scan cycle for the complete gradient The SWATH experiment was set to acquire 100 product ion spectra from *m*/*z* 350 to 1500 per scan cycle with a product ion window set to 6 Da and collision energy from 16 to 60 V with an energy spread of 5 V. The TOF-MS scan acquisition time was set to 50 ms and each product ion scan to 25 ms. The data were acquired and processed using Analyst TF 1.7 software (SCIEX, Canada).

### 4.7. Ion Library Generation

For ion library generation, all mass spectrometry files were searched in unison in ProteinPilot™ software (Version 5.0.2, SCIEX) [85,86] using the paragon algorithm. Samples were uploaded as unlabeled samples using the following parameters: protein identification, digestion with trypsin, and no special factors (for sample preparation). The search was performed by searching a *M. crystallinum* L. genomic database of all *M. crystallinum* ORFs [87]. Peptides with confidence higher than 95% were selected, and the distinct proteins in the final ion library were filtered at a critical false discovery rate of 1%.

### 4.8. Peptide and Protein Quantification

Spectral alignment and targeted data extraction of SWATH-MS data were conducted in the SWATH Processing Micro App in PeakView (Version 1.2, SCIEX) using the spectral library generated above. All raw files were loaded using an extraction window of 15 min with the following parameters: 6 peptides, 5 transitions and peptide confidence of >95%, excluded shared peptides, and extracted ion chromatogram (XIC) width set at 75 ppm. The peak area of proteins from all samples was normalized to protein concentration measured at O.D._280_.

### 4.9. Trans Membrane Domain, Functional Annotation and Subcellular Localisation

Transmembrane domains of identified proteins were predicted using the transmembrane topology (HMMTOP v2.0 and TMHMM v2.0) and beta-barrel membrane (MCMBB and TMBETADISC-RBF) prediction programs to describe the composition of the *M. crystallinum* membrane proteome [33,34,35,36]. Functional annotation of the identified *M. crystallinum* proteome was based on the best BLASTP hits found in the National Center for Biotechnology Information (NCBI), UniprotKB/SwissProt *Arabidopsis thaliana* database (May 2020), with 10^−5^ as the e-value cut-off. *Arabidopsis thaliana* genome accessions (AGIs) of the matched proteins were then submitted to Subcellular Localisation Database For *Arabidopsis* Proteins 4 (SUBA4) [37] for subcellular location annotation.

## 5. Conclusions and Perspectives

The future of plant biology studies is the progression from the mere identification of biomolecules in a given organelle to the functional characterization of biomolecular properties in complex cellular environments, that is, associating the results of multiple “omics” studies to help explain the physiological mechanism in plants under different conditions. Relying on the distinct surface charges instead of buoyant densities, this study highlights the advantages of FFE separation technology to provide a means to simultaneously isolate multiple subcellular membranes at high resolution without the need for initial organelle purification, to give insight into protein function and provide valuable information on proteins located in multiple cellular compartments. Due to the non-denaturing feature of the FFE technique, fractionated microsomal membrane samples assigned to known subcellular compartments can also be used for lipidomic studies in the future, and with the knowledge of the abundance and locations of lipid metabolism-related proteins characterized in this study, a sophisticated model for plant membrane metabolism under various developmental and environmental conditions may be achieved.

## Figures and Tables

**Table 1:** Digital western FFE profiles of selected membrane markers from different subcellular compartments ^a^.

Uniprot Recommended Name (Gene) ^b^	Location ^c^	Digital Western FFE Profiles ^d^
		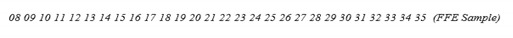
V-type proton ATPase subunit a3 (VHA-a3)	TP	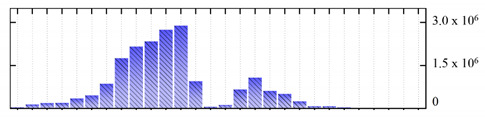
aquaporin TIP1-1/gamma-TIP (TIP1-1)	TP	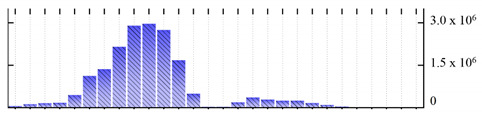
very-long-chain 3-oxoacyl-CoA reductase 1 (KCR1)	ER	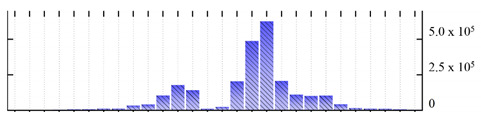
cytochrome b5 isoform B (CYTB5-B)	ER	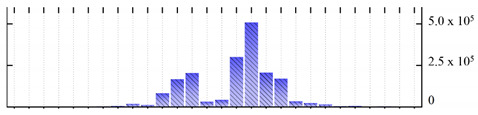
polygalacturonate 4-alpha-galacturonosyltransferase (GAUT1)	GA	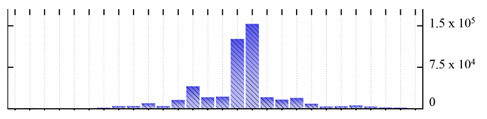
galacturonosyltransferase 9 (GAUT9)	GA	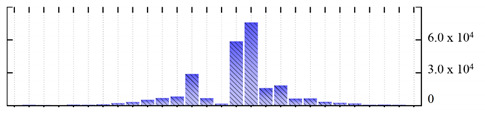
translocase of chloroplast 110 (TIC110)	Chl	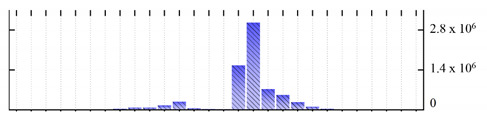
chlorophyll a-b binding protein 1 (LHCB1.3)	Chl	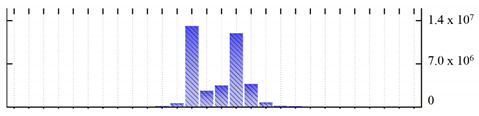
mitochondrial outer membrane protein porin 2 (VDAC2)	MT	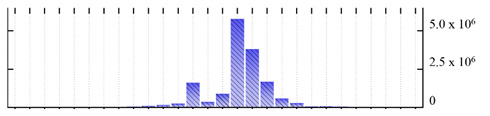
prohibitin-3 (PHB3)	MT	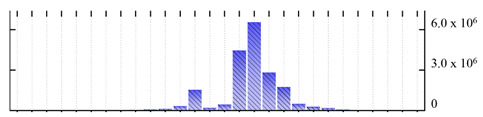
ATPase 2, plasma membrane-type (AHA2)	PM	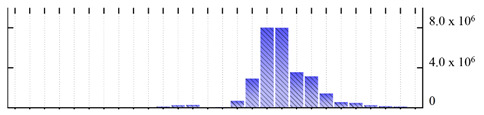
MipA (mipA)	PM	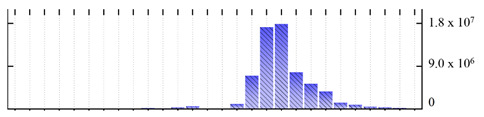

^a^ Digital western FFE profiles were generated based on the peak area from SWATH-MS quantification; ^b^ Sequences of *M. crystallinum* proteins were BLAST in NCBI against the UniprotKB/Swissprot *A. thaliana* database; descriptions are based on UniprotKB/Swissprot *A. thaliana* annotations; ^c^ Subcellular location predictions by SUBAcon. TP: tonoplast, ER: endoplasmic reticulum, GA: Golgi apparatus, Chl: chloroplast, MT: mitochondria, PM: plasma membrane; ^d^ The *x*-axis of FFE profiles indicates FFE samples (8–35), the *y*-axis indicates the average relative abundance (peak area) of three biological replicates for the proteins in the corresponding FFE sample.

**Table 2:** Digital western FFE profiles of tonoplast marker proteins ^a^.

Uniprot Recommended Name (Gene) ^b^	Digital Western FFE Profiles ^c^
	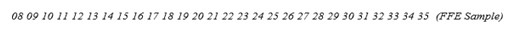
V-type proton ATPase subunit A (VHA-A)	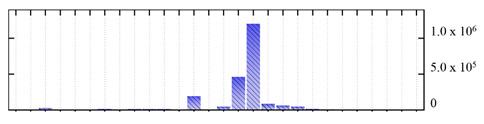
V-type proton ATPase subunit B2 (VHA-B2)	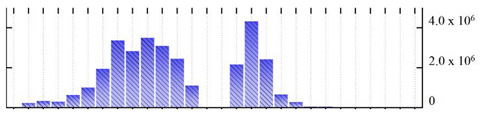
V-type proton ATPase subunit C (VHA-C)	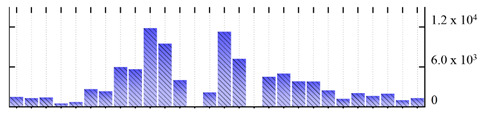
V-type proton ATPase subunit D (VHA-D)	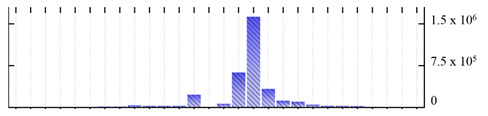
V-type proton ATPase subunit E3 (VHA-E3)	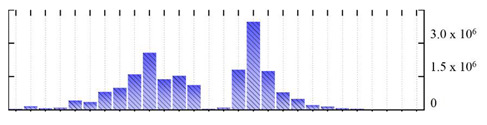
V-type proton ATPase subunit F (VHA-F)	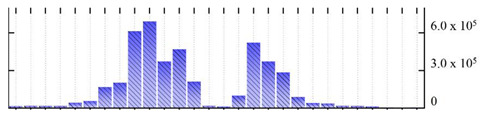
V-type proton ATPase subunit G1 (VHA-G1)	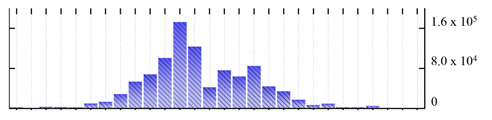
V-type proton ATPase subunit H (VHA-H)	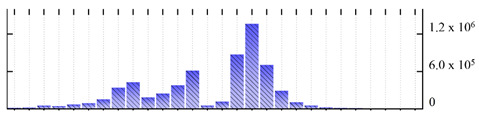
V-type proton ATPase subunit a3 (VHA-a3)	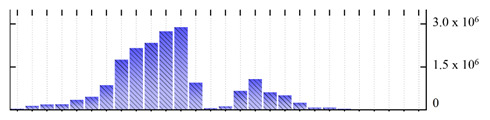
V-type proton ATPase subunit c2 (VHA-c2)	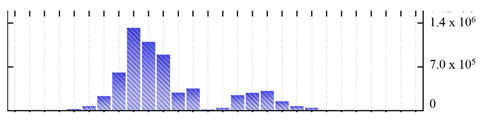
V-type proton ATPase subunit d2 (VHA-d2)	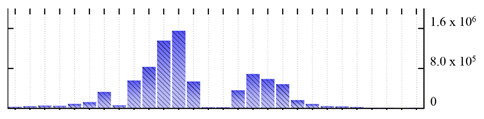
pyrophosphate-energized vacuolar membrane proton pump 1 (AVP1)	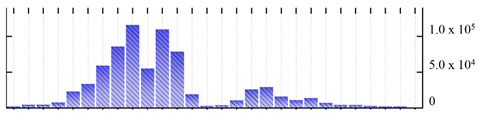
aquaporin TIP2-1/Delta-TIP (TIP2-1)	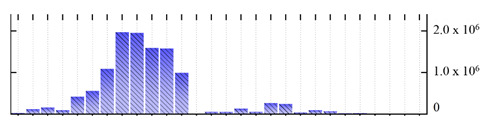
aquaporin TIP1-1/gamma-TIP (TIP1-1)	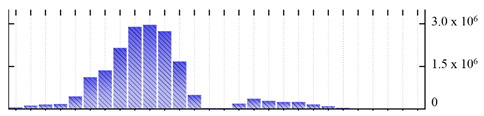
two-pore calcium channel protein 1 (TPC1)	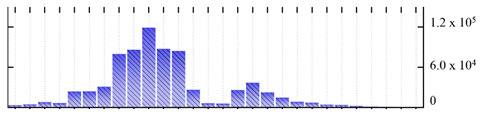
chloride channel protein CLC-b (CLC-B)	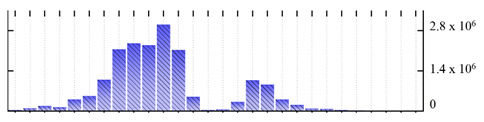
chloride channel protein CLC-c (CLC-C)	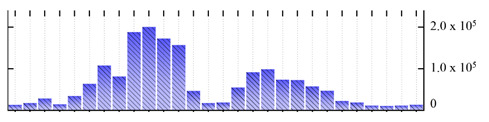

^a,b,c^ Footnotes are the same as for Table 1.

**Table 3:** Digital western FFE profiles of markers of cargo transport-involved subcellular compartments ^a^.

Uniprot Recommended Name (Gene) ^b^	Location ^c^	Digital Western FFE Profiles ^d^
		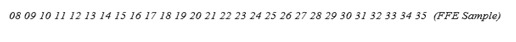
calreticulin-1 (CRT1)	ER	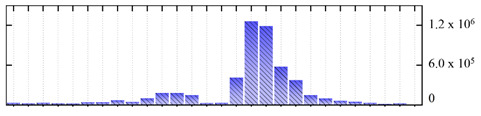
SEC12-like protein 2 (STL2P)	ER	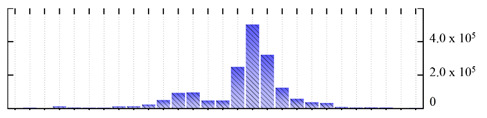
protein glycosyltransferase subunit 1B (OST1B)	ER	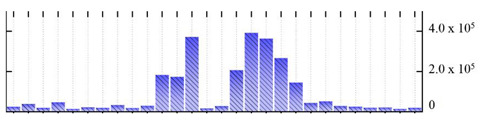
translocon-associated protein subunit alpha (At2g21160)	ER	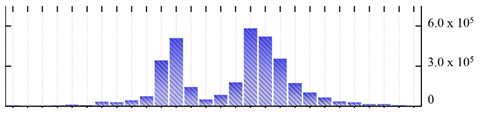
heat shock 70 kDa protein BIP2 (BIP2)	ER	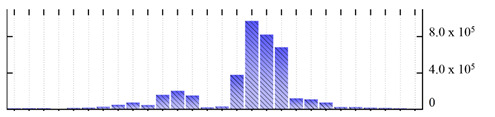
ADP-ribosylation factor 1 (ARF1)	ER-GA	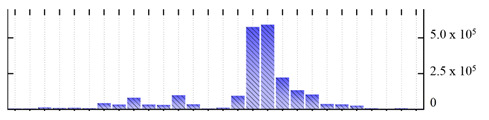
coatomer subunit alpha-1 (At1g62020)	ER-GA	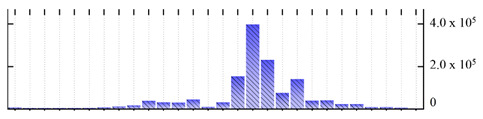
coatomer subunit beta-1 (At4g31480)	ER-GA	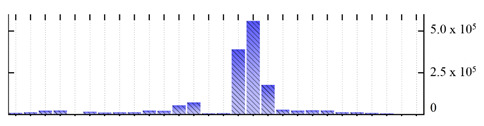
coatomer subunit beta’-2 (At1g52360)	ER-GA	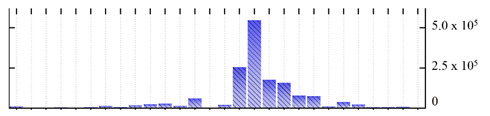
coatomer subunit gamma (At4g34450)	ER-GA	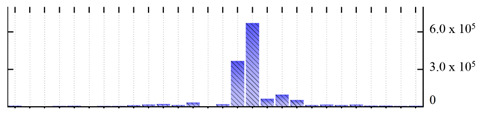
transport protein Sec23/24-like (At3g07100)	ER-GA	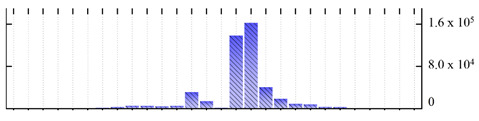
apyrase 2 (APY2)	GA	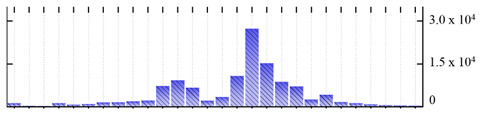
polygalacturonate 4-alpha-galacturonosyltransferase (GAUT1)	GA	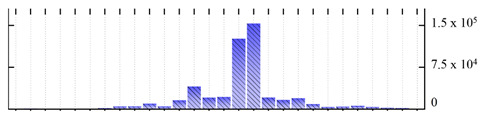
galacturonosyltransferase 9 (GAUT9)	GA	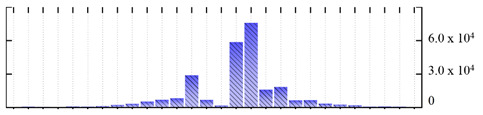
probable methyltransferase PMT8 (At1g04430)	GA	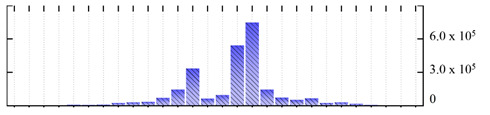
AP-1 complex subunit gamma-2 (At1g60070)	TGN	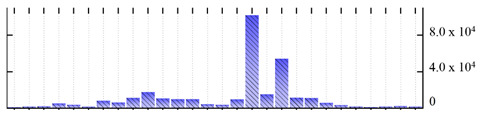
clathrin heavy chain 1 (CHC1)	TGN	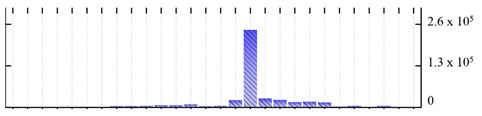

^a,b,d^ Footnotes are the same as for Table 1; ^c^ Subcellular location abbreviations: ER, endoplasmic reticulum; ER-GA, protein exchange between ER and Golgi; GA, Golgi apparatus; TGN, trans-Golgi network.

**Table 4:** Digital western FFE profiles of markers localized to chloroplast envelope and thylakoid membranes ^a^.

Uniprot Recommended Name (Gene) ^b^	Location ^c^	Digital Western FFE Profiles ^d^
		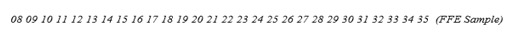
translocase of chloroplast 55 (TIC55)	IME	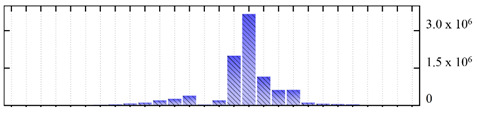
translocase of chloroplast 110 (TIC110)	IME	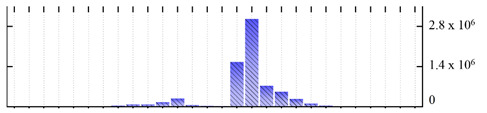
translocase of chloroplast 34 (TOC34)	OME	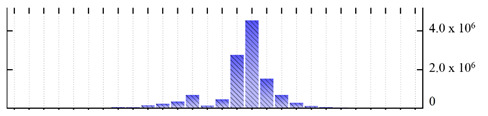
thylakoid lumenal 16.5 kDa protein (At4g02530)	TLK	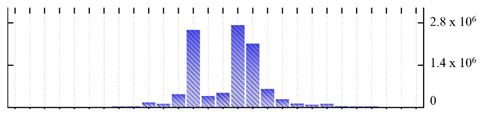
ATP synthase gamma chain 1 (ATPC1)	TLK	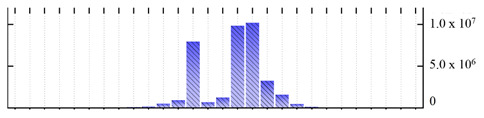
ATP synthase subunit delta (ATPD)	TLK	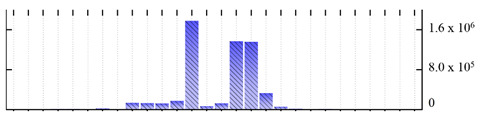
chlorophyll a-b binding protein 1 (LHCB1.3)	TLK	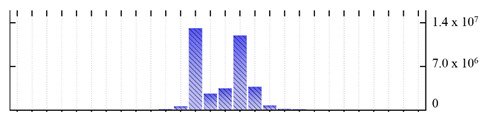
chlorophyll a-b binding protein 6 (LHCA1)	TLK	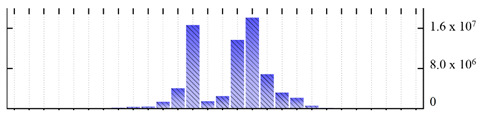
photosystem I reaction center subunit II-2 (PSAD2)	TLK	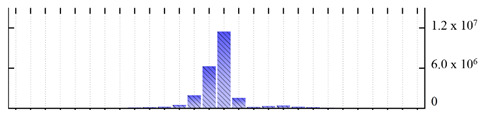
photosystem I reaction center subunit III (PSAF)	TLK	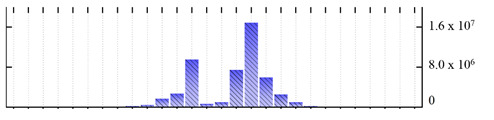
photosystem I reaction center subunit VI-2 (PSAH2)	TLK	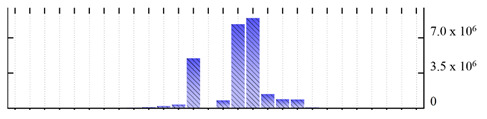
photosystem I reaction center subunit XI (PSAL)	TLK	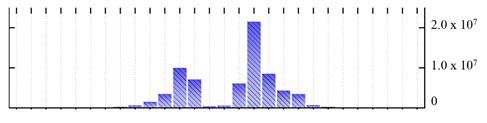
photosystem I reaction center subunit psaK (PSAK)	TLK	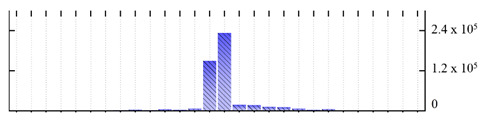
photosystem II stability/assembly factor HCF136 (HCF136)	TLK	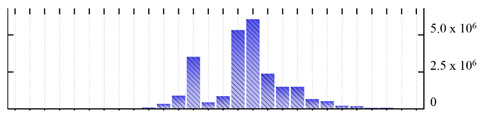
photosystem II 10 kDa polypeptide (PSBR)	TLK	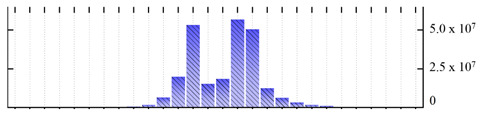

^a,b,d^ Footnotes are the same as for Table 1; ^c^ Subcellular location abbreviations: IME/OME, chloroplast inner/outer envelope; TLK, thylakoid.

**Table 5:** Digital western FFE profiles of mitochondrial markers ^a^.

Uniprot Recommended Name (Gene) ^b^	Location ^c^	Digital Western FFE Profiles ^d^
		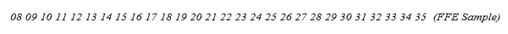
mitochondrial outer membrane protein porin 1 (VDAC1)	MOM	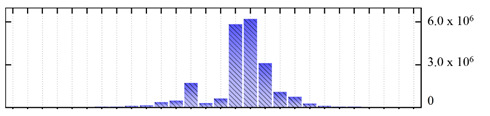
mitochondrial outer membrane protein porin 2 (VDAC2)	MOM	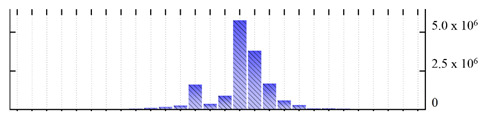
mitochondrial outer membrane protein porin 4 (VDAC4)	MOM	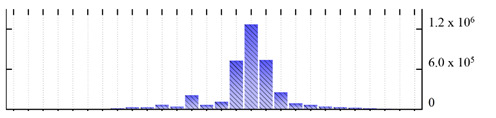
mitochondrial import receptor subunit TOM9-2 (TOM9-2)	MOM	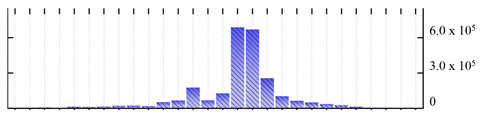
prohibitin-2 (PHB2)	MIM	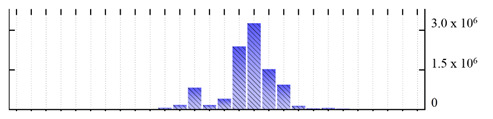
prohibitin-3 (PHB3)	MIM	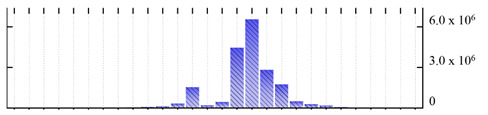
ATP synthase subunit beta-3 (At5g08680)	MIM	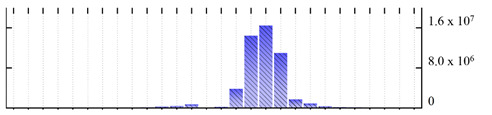
cytochrome b-c1 complex subunit rieske-1 (UCR1-1)	MIM	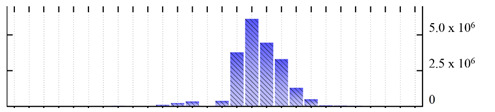
cytochrome c oxidase subunit 5b-2 (COX5B-2)	MIM	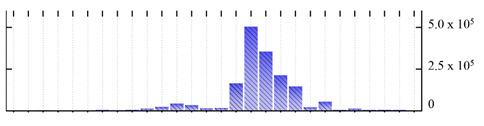
mitochondrial-processing peptidase subunit beta (MPPbeta)	MIM	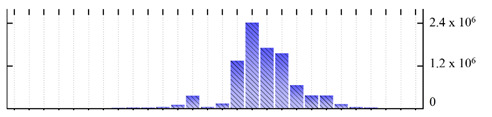
mitochondrial carnitine/acylcarnitine carrier-like protein (BOU)	MIM	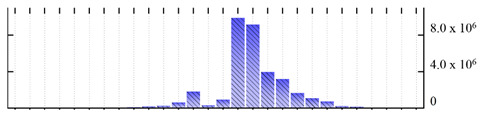
mitochondrial phosphate carrier protein (MPT3)	MIM	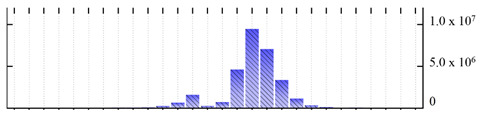
mitochondrial dicarboxylate/tricarboxylate transporter DTC (DTC)	MIM	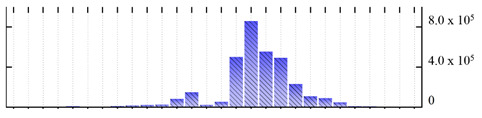
alternative NAD(P)H-ubiquinone oxidoreductase C1 (NDC1)	DL	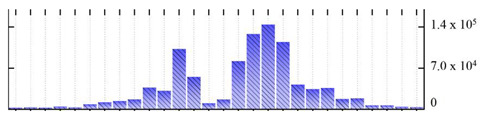

^a,b,d^ Footnotes are the same as for Table 1; ^c^ Subcellular location abbreviations: MIM/MOM, mitochondrial inner/outer membrane; DL, protein dual-localized to chloroplast and mitochondrial membranes.

**Table 6:** Digital western FFE profiles of plasma membrane markers ^a^.

Uniprot Recommended Name (Gene) ^b^	Digital Western FFE Profiles ^c^
	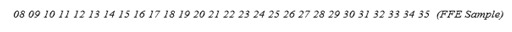
ATPase 2, plasma membrane-type (AHA2)	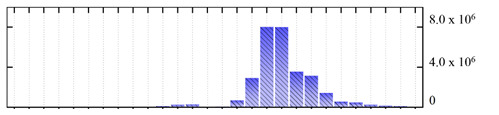
ATPase 4, plasma membrane-type (AHA4)	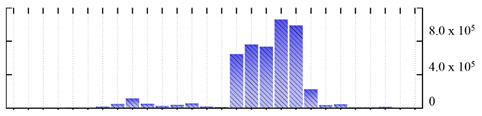
ATPase 5, plasma membrane-type (AHA5)	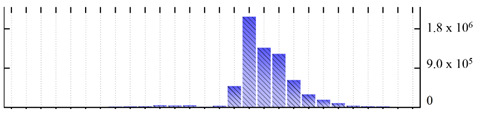
MipA (mipA)	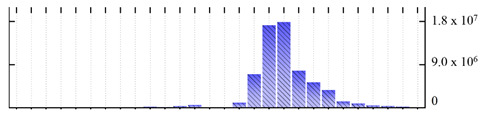
MipD (mipD)	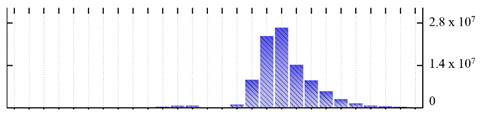
MipH (mipH)	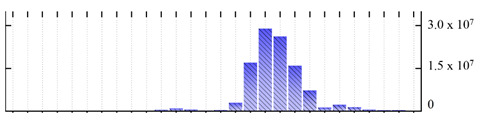
aquaporin pip2;5 (PIP2-5)	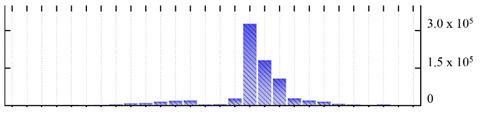
aquaporin pip2;8 (PIP2-8)	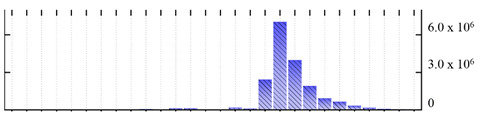
CSC1-like protein ERD4 (ERD4)	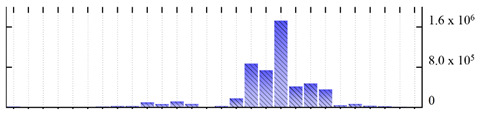
sucrose transport protein SUC2 (SUC2)	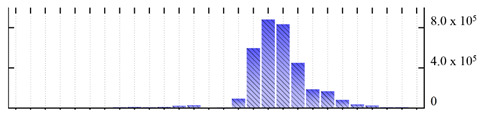
polyol transporter 5 (PLT5)	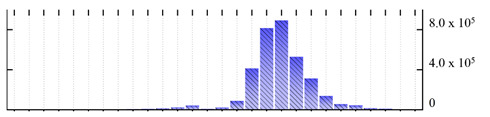
fasciclin-like arabinogalactan protein 2 (FLA2)	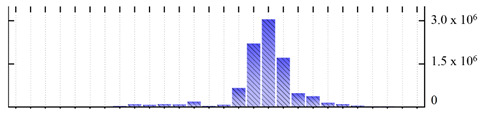
fasciclin-like arabinogalactan protein 6 (FLA6)	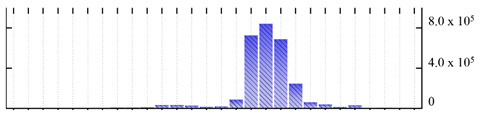
early nodulin-like protein 2 (At4g27520)	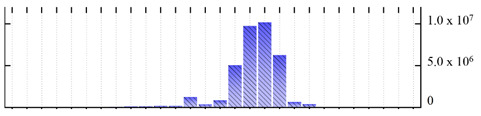

^a,b,c^ Footnotes are the same as for Table 1.

**Table 7:** Unexpected FFE profiles of proteins known to localize to plasma membrane ^a^.

Uniprot Recommended Name (Gene) ^b^	Digital Western FFE Profiles ^c^
	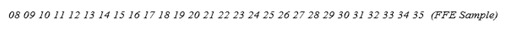
sodium/hydrogen exchanger 7 (NHX7)	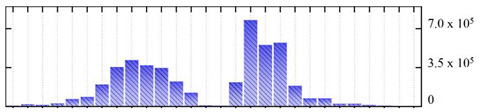
sodium/calcium exchanger (NCL)	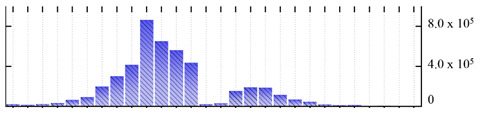
ammonium transporter 1 member 1 (AMT1-1)	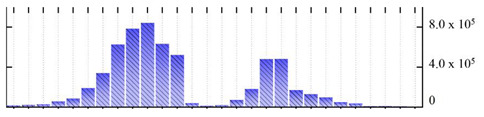
sulfite exporter TauE/SafE family protein 4 (TauE)	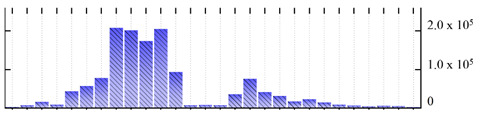
ABC transporter G family member 40 (ABCG40)	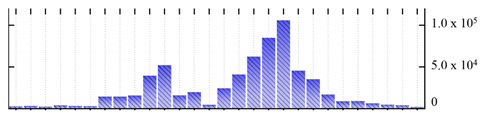
ABC transporter C family member 4 (ABCC4)	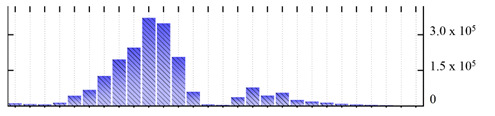
equilibrative nucleotide transporter 1 (ENT1)	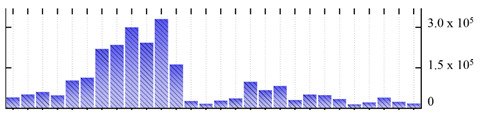

^a,b,c^ Footnotes are the same as for Table 1.

**Table 8:** Digital western FFE profiles of lipid metabolism-related proteins ^a^.

Uniprot Recommended Name (Gene) ^b^	Lipid Metabolism Pathway ^c^	Location ^d^	Digital Western FFE Profiles ^e^
			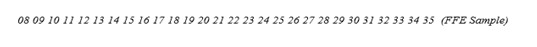
sterol methyltransferase 1 (SMT1)	sterol synthesis	GA	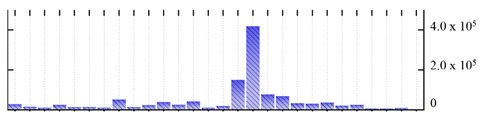
glycerol-3-phosphate acyltransferase 4 (GPAT4)	suberin synthesis and transport	GA	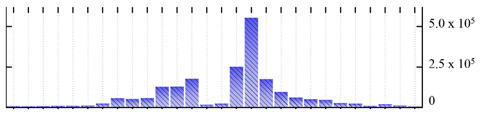
3-ketoacyl-CoA synthase 10 (FDH)	fatty acid synthesis	GA	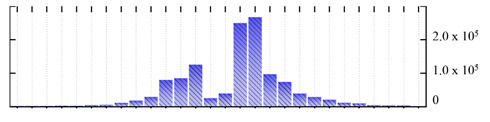
very-long-chain 3-oxoacyl-CoA reductase 1 (KCR1)	fatty acid elongation and wax biosynthesis	ER	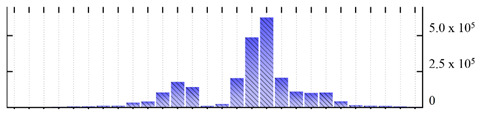
cinnamate-4-hydroxylase (CYP73A5)	fatty acid elongation and wax biosynthesis	ER	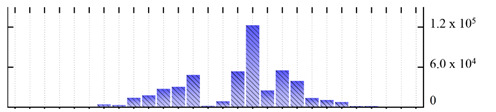
cytochrome b5 isoform B (CYTB5-B)	fatty acid synthesis	ER	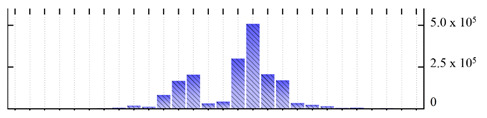
phospholipid: diacylglycerol acyltransferase (PDAT1)	triacylglycerol biosynthesis	ER	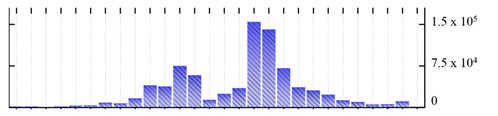
membrane-associated progesterone-binding protein 3 (MSBP2)	steroid binding	ER	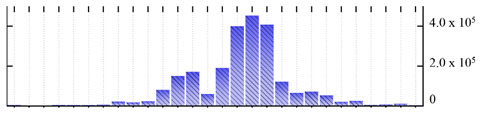
7-dehydrocholesterol reductase (DWF5)	sterol synthesis	ER	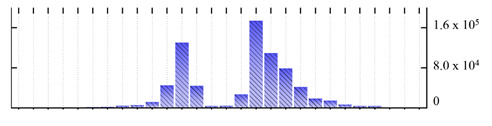
very-long-chain enoyl-CoA reductase (ECR)	fatty acid elongation and wax biosynthesis	ER	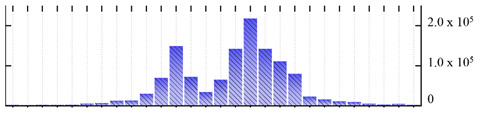
synaptotagmin-1 (SYT1)	lipid binding	ER,PM	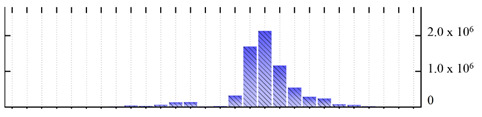
mitochondrial acyl carrier protein 2 (ACP)	mitochondrial fatty acid and lipoic acid synthesis	MT	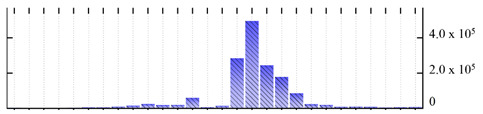
non-specific phospholipase C4 (NPC4)	eukaryotic galactolipid and sulfolipid synthesis	PM	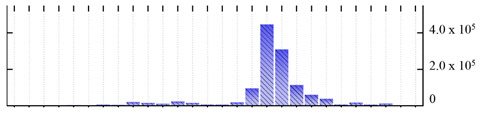
PLC-like phosphodiesterases superfamily protein (At5g67130)	lipid signaling	PM	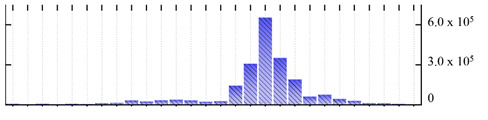
phospholipase D delta (PLDDELTA)	phospholipid signaling	PM	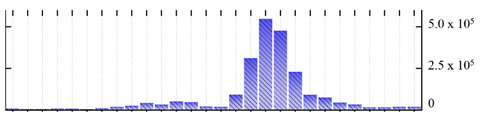
non-specific lipid transfer protein GPI-anchored 2 (LTPG2)	fatty acid elongation and wax biosynthesis	PM	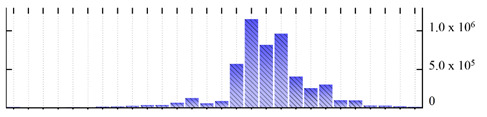
phosphatidylinositol-specific phospholipase C4 (PLC4)	phospholipid signaling	PM	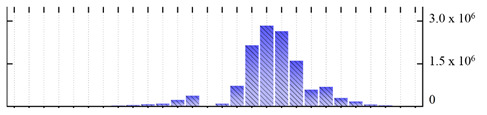
phosphatidylinositol 3- and 4-kinase family protein (PI4KA1)	phospholipid signaling	PM	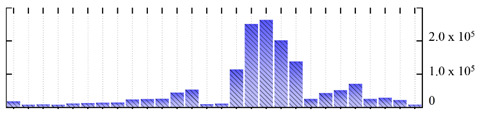
AMP-dependent synthetase and ligase family protein (LACS4)	eukaryotic phospholipid synthesis and editing	PM	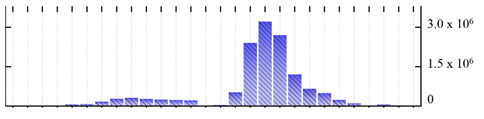
delta(14)-sterol reductase (FK)	sterol synthesis	PM	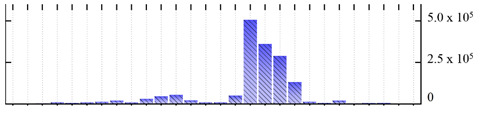
3-ketoacyl-CoA synthase 6 (CUT1)	fatty acid elongation and wax biosynthesis	PM	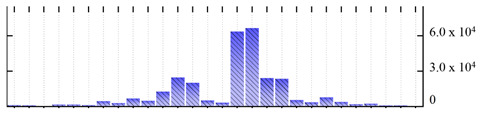
ABC-2 type transporter family protein (ABCG15)	lipid transport	PM	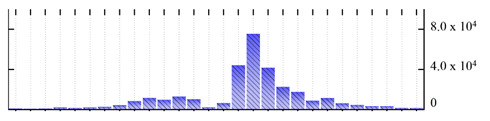
ABC-2 type transporter family protein (ABCG22)	lipid transport	PM	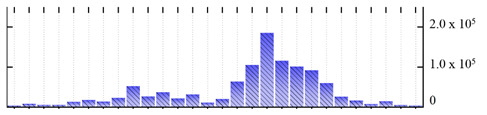
esterase lipase thioesterase family protein (At1g54570)	fatty acid elongation and wax biosynthesis	Chl	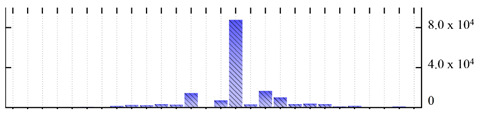
trigalactosyldiacyl-glycerol 2 (TGD2)	eukaryotic galactolipid and sulfolipid synthesis	Chl	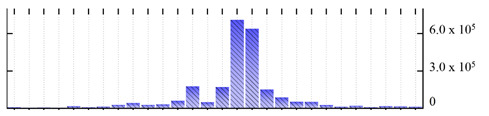
trigalactosyldiacyl-glycerol 4 (TGD4)	eukaryotic galactolipid and sulfolipid synthesis	Chl	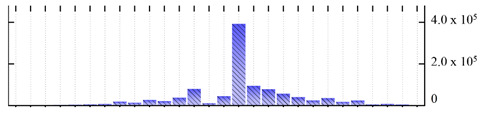
translocon at the outer envelope membrane of chloroplasts 159 (TOC159)	galactolipid, sulfolipid, and phospholipid synthesis	Chl	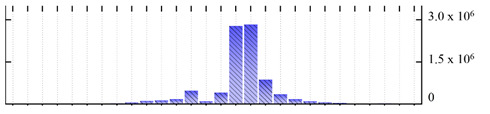
alpha beta-hydrolases superfamily protein (DALL3)	oxylipin metabolism	Chl	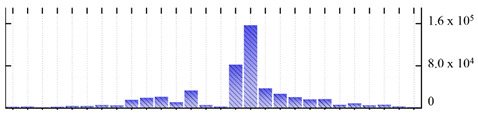
single hybrid motif superfamily protein (BCCP2)	fatty acid synthesis	Chl	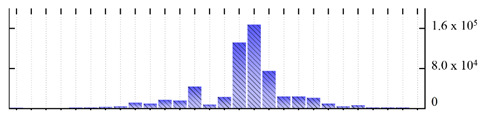
long-chain acyl-CoA synthetase 9 (LACS9)	fatty acid synthesis galactolipid, sulfolipid, and phospholipid synthesis	Chl	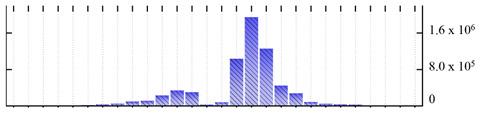
fatty acid desaturase 6 (FAD6)	fatty acid desaturation galactolipid, sulfolipid, and phospholipid synthesis	Chl	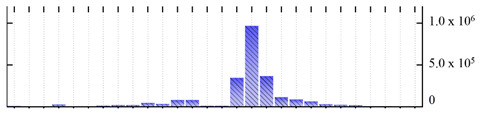
fatty acid desaturase 8 (FAD8)	fatty acid desaturation galactolipid, sulfolipid, and phospholipid synthesis	Chl	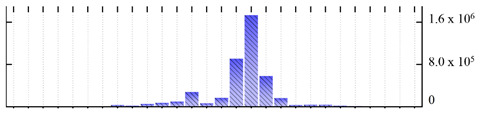
phospholipid glycerol acyltransferase family protein (ATS1/GPAT)	galactolipid, sulfolipid, and phospholipid synthesis	Chl	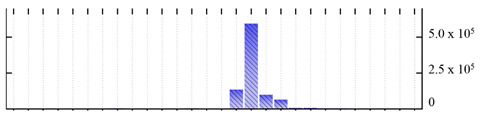
AMP-dependent synthetase and ligase family protein (AAE16)	fatty acid metabolism	Chl	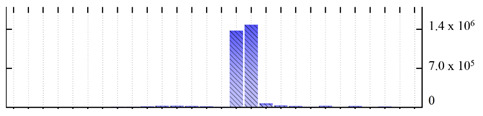
acetyl CoA carboxylase carboxyltransferase alpha subunit (CAC3)	fatty acid synthesis	Chl	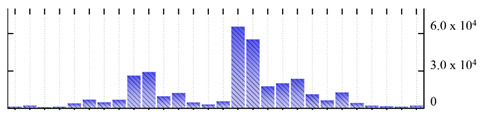

^a,b,d,e^ Footnotes are the same as for Table 1; ^c^ Lipid metabolism pathways were assigned based on the *A. thaliana* Acyl-Lipid Metabolism Database [51] and Uniprot annotations.

**Table 9:** Protein profile of ferredoxin compared with three other thylakoid markers ^a^.

Uniprot Recommended Name ^b^	Digital Western FFE Profiles ^c^
	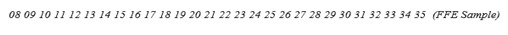
ferredoxin-1 (FD1)	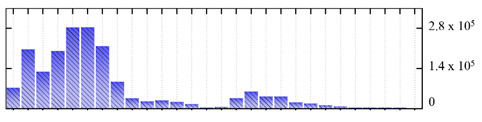
ATP synthase gamma chain 1 (ATPC1)	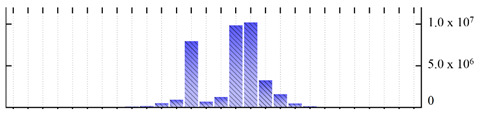
ATP synthase subunit delta (ATPD)	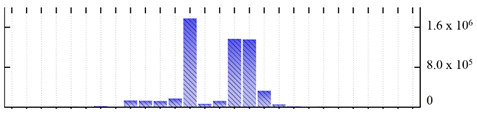
photosystem I reaction center subunit VI-2 (PSAH2)	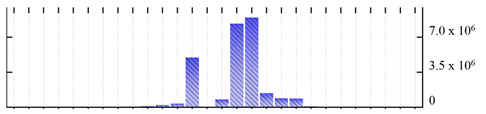

^a,b,c^ Footnotes are the same as for Table 4.

## Data Availability

Raw spectral data were submitted to the Southern Cross University research portal (https://researchportal.scu.edu.au/, accessed on 4 May 2021) and are available under DOI:10.25918/data.134.

## Supplementary Materials

The following are available online at https://www.mdpi.com/article/10.3390/ijms22095020/s1, Table S1: Transmembrane predictions of identified proteins by four different programs, Table S2: Functional annotation and subcellular localization of identified proteins, Table S3: Digital western FFE profiles of Ras-related proteins.
